# Long COVID as a never-ending puzzle: the experience of primary care physicians. A qualitative interview study

**DOI:** 10.3399/BJGPO.2023.0074

**Published:** 2023-11-01

**Authors:** Danica Rotar Pavlic, Alem Maksuti, Matic Mihevc, Ana Munda, Karmen Medija, Viktor Strauch

**Affiliations:** 1 Galenia d.o.o, Ljubljana, Slovenia; 2 Faculty of Medicine, University of Ljubljana, Ljubljana, Slovenia; 3 Persuasion d.o.o, Ljubljana, Slovenia; 4 Primary Healthcare Research and Development Institute, Health Centre Ljubljana, Ljubljana, Slovenia; 5 University Clinical Center Ljubljana, Ljubljana, Slovenia

**Keywords:** long COVID, post-acute COVID-19 syndrome, primary healthcare, qualitative research, Slovenia

## Abstract

**Background:**

Long COVID provides a new context in which primary health care needs to be re-examined, especially because it has health and social dimensions. Primary care physicians’ experiences and perceptions of caring for patients with long COVID are an underexplored area.

**Aim:**

To explore the experiences of Slovenian primary care physicians in management and treatment of patients with long COVID.

**Design & setting:**

A qualitative interview study of physicians in Slovenian primary care.

**Method:**

Semi-structured interviews were held with physicians who had treated patients with long COVID until saturation was reached. The interviews were carried out between November 2021 and April 2022. Qualitative content analysis (QCA) was used to analyse the data collected.

**Results:**

Seventeen participants were interviewed. The following five categories were defined based on the coding process: the definition and symptoms of long COVID; social exclusion; sick leave and returning to the work environment; cooperation with rehabilitation centres; and the importance of trust and good communication with the patient.

**Conclusion:**

The study showed the experiences of Slovenian primary care physicians in the management and treatment of long COVID. The problems related to long COVID were divided into two groups: health problems and psychosocial problems. Slovenian physicians have the greatest problems with dealing with the patient’s ability to work. It was found that adequate communication and trust between physicians and patients are two important indicators for an integrated model of managing long COVID.

## How this fits in

Long COVID is a complex condition that persists after COVID-19 infection, causing diverse physical and psychological symptoms. Although its medical aspects are extensively studied, the psychosocial implications require further exploration. Insights from Slovenian GPs, who faced prolonged lockdowns and pandemic challenges, highlighted the importance of effective communication and trust in addressing the impact of long COVID on patients' work and personal life. Facilitating patients’ return to work is crucial for recovery, with a behavioural approach considering psychosocial factors contributing to better outcomes.

## Introduction

One of the enduring repercussions of the COVID-19 pandemic, which has persisted for the past 3 years, is a phenomenon referred to as 'long COVID' or 'long-haulers' within the medical community. This is a post-COVID condition defined as an umbrella term for a complex multisystem illness.^1^ Cook *et al* noted that long COVID is related to a variety of diseases and symptoms, such as fatigue, dyspnoea, cardiovascular abnormalities, mental health problems, olfactory and gustatory dysfunction, and pulmonary symptoms.^2^ Estimates of the prevalence of long COVID vary from 10% to 35%, although the true extent of its occurrence is not yet fully understood.^3^ The literature contains several views of medical researchers that offer various estimates of the actual duration of long COVID.^4^ National Institute for Health and Care Excellence,^5^ Scottish Intercollegiate Guideline Network,^6^ and the Royal College of General Practitioners^7^ have established comprehensive definitions, norms, and guidelines to address the multifaceted nature of long COVID.^8^ However, the reality faced by patients and medical staff is complex because of sometimes controversial clinical conditions. Above all, studies of survivors and physicians report a wide array of experiences among individuals who have been struggling with post-COVID symptoms for multiple years.^9–12^

Each qualitative study dealing with people’s experiences is a piece of the puzzle in understanding such a complex field. Particularly noteworthy are case studies conducted within specific countries. These studies offer better insight into the context of what is happening in a country during the COVID-19 pandemic.^12,13^ At the same time, these same studies are a good basis for comparison and identifying common experiences across different countries.

Although there is consensus among medical professionals regarding the medical aspects of long COVID, there are variations when it comes to its psychosocial dimension within the context of the COVID-19 pandemic. This psychosocial dimension encompasses not only the physical health challenges faced by individuals but also the impacts on other facets of their lives, such as diminished self-esteem, feelings of being stigmatised owing to the disease, and negative effects on family relationships.^3,14^ The goal of the research was to determine the views of primary care physicians because several dilemmas arose in the field regarding the care of patients with long COVID; research that included the views and experiences of patients has already been conducted.^15^

Qualitative studies involving primary care physicians and nurses offer valuable insights.^16,17^ These healthcare professionals have a unique advantage because they observe their patients over an extended period, which provides them with deep insights into their patients’ health.^18^ Simultaneously, they also know their patients’ social circumstances, making them an ideal source of information about how people cope with the consequences of long COVID, both medically and otherwise, including their own experiences with COVID.^17^

This qualitative study focused on the experiences of primary care physicians in Slovenia in the context of long COVID. The aim of this qualitative study was to provide '*understanding with respect to decisions and behaviours of patients and professionals'*;^19^ specifically, primary care physicians dealing with long COVID in Slovenia.

The study proceeded from the assumption that the experiences of Slovenian primary care physicians are useful material for a qualitative analysis, given that the country experienced one of the longest periods of lockdown and problems in pandemic control.^20^ The research question was the following: What are the experiences of primary care physicians when it comes to patients who have recovered from COVID-19 and now face the consequences of long COVID? The investigation focused on patients’ health problems as well as problems related to their reintegration into the social and work environment.

## Method

### Sampling strategy and recruitment

This study was carried out at the University of Ljubljana’s Medical Faculty from November 2021–April 2022. The data collection involved in-depth interviews administered by seven doctoral students with a special interest in qualitative methodology. The in-depth interviews fostered rich interaction between the interviewees and the interviewers, allowing for comprehensive exploration of the subject matter.^21^ The topic guide was developed in November 2021. Because long COVID has not been fully explored, the study used a semi-structured questionnaire, which allowed the interviewee to provide answers that were not initially expected. This gave the study greater heuristic potential.

The research was carried out during the period of the COVID-19 pandemic. All research was more difficult to carry out during this period. Participants were recruited from the database of the Association of Family Medicine Physicians. Participants were made aware of the study by a further explanatory text, which was approved by the Slovenian Republic Commission for Medical Ethics. The researchers were not involved in the participants’ direct care. The informed consent form provided information on the purpose of the research, and a confidentiality statement of how participant information would be securely handled was obtained by each participant. It was possible to include primary care physicians with different characteristics from the whole of Slovenia. Primary healthcare physicians were selectively chosen to include a sample of physicians working in rural and urban areas, as well as in public and private healthcare centres (Table 1). The sample size was determined based on a saturation process. The data collection process stopped when new data no longer offered extra insight into the research question.^22^ Data saturation was achieved after 17 interviews. At the end of the interview, each participant was asked if they wished to receive a summary of the findings in the form of a general summary and/or a publication arising from the study. No compensation was offered for their time.

**Table 1.** Participant characteristics

Participant number	Sex	Age, years	Area of practice	Years of service	Institution of employment
GP1	F	52	Rural	26	HC and MF
GP2	F	63	Rural	37	HC
GP3	M	33	Urban	6	HC
GP4	M	41	Urban	9	Concessionaire
GP5	M	63	Urban	37	Concessionaire
GP6	M	38	Urban	14	HC
GP7	F	42	Urban	14	HC
GP8	F	58	Urban	32	Concessionaire
GP9	M	60	Urban	31	Concessionaire
GP10	F	45	Urban	20	HC and MF
GP11	F	51	Urban	25	HC
GP12	F	61	Urban	32	HC
GP13	F	35	Rural	7	HC
GP14	F	46	Rural	20	HC
GP 15	F	33	Rural	7	HC
GP16	F	32	Rural	5	Concessionaire
GP17	F	48	Urban	19	Concessionaire

HC = health centre. MF = medical faculty.

### Data analysis

All interviews were recorded and transcribed verbatim. Qualitative content analysis was used —specifically, inductive thematic analysis^23^ — to analyse the data collected. The main feature of this technique is that extensive texts are classified into smaller content categories. This technique contains an early phase of preparation and organisation, including open coding, category formation, and abstraction.

Seven doctoral students independently coded the interviews. The training for coders was held by DRP and AM at the University of Ljubljana’s Department of Family Medicine on 22 November 2021. DRP and AM, as the main researchers, led the group and reviewed recordings, transcripts, and codes; in March and June 2022, they carried out checks in cases of discrepancies. The coders used a manual coding technique.^24^ After the independent coding process was concluded, a final codebook was developed through a triangulation process among the coders. If consensus was not attained, there was an attempt to achieve intercoder agreement on the differently perceived parts of the text analysed to fit the category created, also known as the 'unitising' process.^25^

The results of the analysis are presented in accordance with the standards for reporting qualitative research (SRQR).^26^ The resulting categories are explained, and specific examples are given with quotes from the interviews.

## Results

The study included 17 primary care physicians from across Slovenia. The sample included physicians working at health centres as well as independent concessionaires or contractors from urban and rural areas (that is, physicians who have been granted a concession by the Ministry of Health to provide health services independently within the public health system). Their sociodemographic data are presented in Table 1.

The interviews lasted between 35 and 90 minutes. Based on data analysis, the following five categories were established: (1) the definition and symptoms of long COVID; (2) social exclusion; (3) sick leave, and returning to the work environment; (4) cooperation with rehabilitation centres; and (5) the importance of trust and good communication with the patient.

### Definition and symptoms of long COVID

The results of the study indicated the lack of a single (holistic) definition of long COVID. Although the term is used in medical literature and in clinical practice, practically all interviewees stated that they had difficulties in establishing a diagnosis of long COVID. In doing so, they resorted to the literature, but mostly they helped themselves with the patient’s symptoms.

Another important indicator of the definition of long COVID was the duration of patients’ problems. The interviewees indicated that patients were diagnosed with long COVID if the problems manifested for >3 weeks after contracting the virus, and noted that the problems could last for several months or even years. Table 2 shows the dilemmas and problems that physicians perceived in diagnosing and interpreting symptoms.

**Table 2.** The dilemmas and problems that physicians perceived in diagnosing and interpreting symptoms

Theme	Category	Code	Quotes
**Definition and symptoms** **of long COVID**	Understanding the diagnosis	Definition	'*It is about several very different problems, the only connecting moment of which is that the patient's got over COVID.'* *'If I make a diagnosis, then I have to have some proof. But now I have no evidence. We use diagnoses based on the symptoms or signs that the patients state. And then we write in the medical record itself that it could be as a result of COVID.'* *'In the case of COVID, it seems to me that it is about people with medically unexplained conditions. They have more problems that are related to COVID more indirectly through the social environment.'*
	Symptoms may belong to other diseases and circumstances	Fatigue, lack of energy	*'The symptoms are actually very non-specific and we can’t say they’re typical of long COVID. They may be typical of many other diseases resulting from chronic fatigue.'* *'These are patients who have not been to the clinic before. If that were true, you could just say that there was a worsening of the previous problems, but that’s not true. These are people who used to be active, athletes, very fit, but today they don't get back to that condition.'* *'Patients are affected in the sense that they feel very bad and the most frustrating thing for them is that the doctor can't find anything! That is, when the doctor does laboratory tests, X-ray of the lungs, spirometry, when he measures the saturation ... That is, all the vital signs are fine, the whole laboratory is fine, you can't see anything on the X-ray, except maybe the remnants of COVID pneumonia, but the patients they feel bad.'*
		Depression and anxiety	*'Most of these patients were already taking antidepressants. I cannot say that it is a new problem of long COVID, but it is possible that due to COVID there has been a worsening of these depressive or anxiety symptoms.'*
		Prolonged cough	*'It is probably not the infection itself that matters, but everything that followed the infection itself. Some even went to work with a cough, while others did not.'*
		Chronic disease deterioration	*'I see a deterioration of chronic diseases, like asthma and chronic bronchitis, in some patients.'*
		Mixed symptoms	*'They could be very affected patients, almost immobile, needing to learn to walk again, with speech problems due to intubation, they had breathing difficulties, chest pains. Then there is another group of these patients, where the disease itself was not so severe, but they cited all kinds of problems. From heavy breathing, tightness in the chest, rapid fatigue, insomnia, headaches, changes in mood, concentration, hair loss are noticed. A lot of these problems that you can't just attribute to COVID.'*
	Chronological definition	Symptoms develop 3 weeks–3 months after recovery	*'The symptoms appear somewhere around 3 weeks to 3 months after recovery from COVID, independently of other symptoms.'*
		Symptoms develop 10 days after isolation and last several days or weeks	*'A disease originating in COVID, when this doesn’t resolve after 10 days of isolation and continues for days and weeks after recovery from COVID. That is, when the test is already negative.'*
		Symptoms last at least 5–6 weeks after recovery	*'I start thinking it’s long COVID after at least 5–6 weeks.'*
		Based on patients’ descriptions of symptoms	*'No, I still can’t diagnose it as long COVID based on that. I need some kind of evidence to do that. And I still don’t have any evidence at that point.'* *'We simply establish the diagnosis based on the symptoms or signs the patients describe.'*

In seeking to understand why interviewees in this study were particularly challenged and perplexed by the emergence of long COVID, the authors consider the following scenario to be possible: long COVID is a newly emerging concept with no fixed and agreed pathognomic symptoms or signs that can specifically describe this condition; instead, it is a constellation of various symptoms and/or signs. It is possible that patients with medically unexplained symptoms are now being included under the category of 'long COVID' when presenting to primary care, whether or not these symptoms are, ultimately, a result of COVID infection (a causal relationship which cannot easily be determined in the absence of agreed diagnostic tests for long COVID). As such, GPs such as those interviewed in this study are seeing a large number of patients correctly or incorrectly labelled as having long COVID, with a wide variety of non-specific symptoms. Some of these symptoms may not be connected to COVID infection and may therefore be confounding both diagnosis and treatment approaches. In terms of practically helping patients diagnosed with long COVID, GPs lack new approaches to address these situations, and have no algorithms to follow or therapeutic trials to conduct. As such, the interviewees found themselves scrambling to help their patients, questioning themselves on what to do with them, and constantly reviewing and re-evaluating their symptoms, signs, and diagnoses, just like a detective would in a challenging cold case.

### Social exclusion

The statements of the interviewees confirmed that COVID-19 has changed people’s and physicians’ lives to a large extent. Social exclusion, loneliness, working from home, and the hardships of those socially at risk are some of the indicators that point to the social consequences of long COVID:

*'Everything sort of died with COVID. I’d say that was also a long-term consequence of COVID. It will take a while for things to fall back in place again. I don’t know about my colleagues, but what I especially miss is more authentic contact.'* (GP14)

*'In fact, we are quite isolated, because we have our own practice. Sometimes you call someone for something, because we have telephones ...'* (GP 8)

The already long waiting periods in the system have been further complicated by the COVID-19 pandemic. The fact that everything now took place even more slowly, taking into account the strict anti-COVID-19 measures in health care, made the patients even more frustrated:

*'On the part of patients, their expectations are too high, they complain about long waiting times for specialists.'* (GP3)

Physicians shared the opinion that real problems with patients are yet to come and that they often cannot be solved at the level of primary health care, so they turn to specialists, in this case psychiatrists:

*'In the psychological field, there is a huge problem that I don't know how we will solve it. Even with the fact that some of our colleagues and some of the patients* [confronted with the consequences of long COVID-19] *think they are for a psychiatrist. We are not capable of solving these things.'* (GP7)

### Sick leave and returning to the work environment

After 1 month of a patient's absence from work, the physician must send the documentation to the Institute for Health Insurance, which decides on the extension of sick leave. Documentation must be supported by the most measurable results if possible. Regardless of the fact that people had problems, the physician was not always able to define these problems as sufficient criteria that mean the patient is unable to work. This is a completely different situation from any other disease, which can be defined as having a beginning and end:

*'The problem is that you don’t have a reliable report based on which you can justify that the patient has problems, despite the fact that he really does have problems. I can’t write that the patient is tired and I’m asking for an extension of the sick leave for 14 days. After 14 days he’ll still be tired.'* (GP15)

*'The end of treatment/rehabilitation is more predictable for patients that are on long-term leave due to some other illness or injury, but with long COVID you don’t know when the treatment will even end.'* (GP4)

*'Then they say, it’s just that I’m not quite well yet. And I tell them that, unfortunately, they may not be completely well for another 6 months, and that unfortunately we can’t extend the sick leave until they feel completely OK. And this is very problematic. Some people go back to work, but they still don’t have the capacity because they’re still not OK.'* (GP1)

In addition to all the complications with health, people find it difficult to return to the work environment after long COVID. Sometimes it is the work environment that requires adjustments, and sometimes patients just do not feel useful and capable. Some patients lost their job:

*'This week I had a patient with long-term muscle pain after COVID, but it was clear that it was more a result of the psyche, and he already had previous mental problems. Now he has lost his job and this situation complicated everything. With the other lady, there was also a loss of employment and I had the feeling that this was her major problem.'* (GP1)

*'It’s an interesting story of a young programmer, I think he’s 42-years-old, who needed almost 8 months to recover to the same intellectual or cognitive level. The neurologists took over his case; he went to them for treatment. They confirmed progress through testing. He used to work 10, 12, 15 hours a day, and then he only managed 3–4 hours due to poor concentration. After 8 months, however, his condition improved.'* (GP17)

*'However, there are several such people who may not have been satisfied with their job before and for whom the fact that they contracted COVID is a good reason to remain on sick leave.'* (GP7)

### Cooperation with rehabilitation centres

As an insidious disease, long COVID requires long-term treatment, which also includes clinical treatment, hospitalisation, and rehabilitation measures. In this case, the interviewees pointed out some of the problems they faced in their work. In particular, they pointed out the lack of a unified register and guidelines for treating patients with long COVID.

They also emphasised the poor connection between the post-COVID clinic and rehabilitation centres. Sometimes the services of rehabilitation centres are not covered by the insurance company (they are not part of the public health system), which means that the patients themselves have to pay for the rehabilitation, which the interviewees pointed out as a serious problem:

*'I sent the patients to the post-COVID clinic with the hope that rehabilitation would also be offered, but this wasn’t the case. Faster diagnostics and no smart action.'* (GP3)

*'I think that there are self-paying post-COVID rehabilitation-type services at spas and health resorts; the problem is that patients have to pay for it themselves.'* (GP1)

*'There should be some centres where patients could be referred and then they would determine whether it is really post-COVID syndrome or prolonged COVID. Now patients get a bunch of referrals and wait.'* (GP2)

### The importance of trust and good communication with the patient

When managing patients with long COVID, all interviewees pointed out the path of communication between physicians and patients. In this context, the interviewees also talked about an '*alliance*' and the importance of mutual trust:

*'It’s necessary to understand them, it’s necessary to develop an alliance. I approached this according to the principle of relief, alliance, and support. This concept worked best. Trust has to be established so things will get better, but that takes time. If you had an open conversation to make the patients aware that it was worse at the beginning and is getting better now, they were more reasonable.'* (GP1)

*'The basis is that he trusts you, that you really believe him. This is basic. To try my best to show through verbal and non-verbal communication that I believe him that he really has problems.'* (GP9)

Practically all the interviewees pointed out that it is a lengthy process because long COVID is treated in a family physician’s clinic. Trust is therefore crucial because in this case the physician acts not only as a medical professional, but also often as an arbiter between the patient and the employer. Mutual trust was therefore crucial:

*'I think that it’s often the case that patients basically decide for themselves and think that if they can’t do something at home or that they’re tired, that they’re unable to work, then of course you’ll give them sick leave. As far as that’s concerned, we’re the ones that will say whether they go on sick leave or not.'* (GP5)

## Discussion

### Summary

This qualitative study explored the experiences of Slovenian primary care physicians in addressing the management and treatment of long COVID. The interviewees identified two distinct groups of problems associated with long COVID: health problems and social problems, particularly related to absence from work. The study revealed that Slovenian physicians encountered challenges in diagnosing long COVID owing to the lack of a comprehensive definition or guidelines, making it unclear when the disease actually ends. Many issues surrounding patients’ sick leave stem from this ambiguity.

In addition to the actual treatment, physicians also face difficulties in managing the patient’s overall condition, especially when patients return with persistent problems lasting for months or even years. The findings suggest that COVID-19 and long COVID are not typical diseases, but rather a complex constellation of simultaneous challenges that affect not only patients’ health and lives but also their social wellbeing, including their work.

The participants in this study emphasised two important factors that may facilitate rehabilitation of people with long COVID; namely, effective communication and trust between physicians and patients. Based on their insights, these factors are key to successfully navigating the complexities of long COVID, which remains an ongoing puzzle.

### Strengths and limitations

This study is useful for anyone involved in research on long COVID. Because it is an unexplored area in which there are no clear cause-and-effect relationships, long COVID is an ongoing puzzle. Every study, including this one, contributes considerably to developing of a body of knowledge.

This is the first study of its kind to be conducted in Slovenia. It opens an important research discussion regarding long COVID and all of its medical and social dimensions. The study also has practical utility for preparing national guidelines and for rehabilitation centres because it reveals some of the important ways to manage and treat long COVID.

One weakness of the study is the method used. A general limitation is linked to epistemological criteria and validity in qualitative research.^27,28^ Although qualitative studies reveal many characteristics of the phenomenon investigated, large-scale representative quantitative surveys are needed to capture a large amount of data and shed more light on long COVID. On the other hand, the aforementioned weakness is not a true shortcoming because, given the lack of research on long COVID, it is not possible to put forward hypotheses that can be verified on a large number of participants.

### Comparison with existing literature

The results of this study, similar to the results of studies by researchers elsewhere in the world, have indicated the lack of a single holistic definition of long COVID.^1,2,9–13^ A comparative view also revealed that patients face practically the same symptoms all over the world.^2,3^ On the other hand, physicians cope differently in every country, because healthcare systems differ at a national level.^12,13^

Researchers in other parts of the world also attach great importance to the relationship between the physician and patient in treating long COVID, including the authors of two UK studies.^8,9^ Atherton *et al* note that *'the patient–doctor relationship is vital in the management of people with long COVID'*.^16^ Effective communication and ongoing support through follow-up appointments are vital for rehabilitation and patient satisfaction because it was reported that only patients that were offered this type of support felt that they had received satisfactory care.^9,10^ Furthermore, long COVID encompasses a social dimension that often proves more challenging to address than the physical symptoms of the disease itself.^14^

### Implications for research and practice

The study underscores the significance of effective communication and trust between physicians and patients in addressing long COVID, especially considering its social implications.

Slovenian GPs play a crucial role in facilitating patients’ return to work by providing up to 30 consecutive days of sick leave or conveying relevant information in relation to long COVID, assisting the physician appointed by the Health Insurance Institute of Slovenia in determining the need for prolonged sick leave.

Reintegration into the workforce is an important aspect of illness recovery. Research has indicated that individuals who are unable to work for more than 6 months have less than a 50% likelihood of returning to work in any capacity.^29^ Therefore, when assessing a worker’s fitness for work, it is vital to consider a behavioural approach rather than solely relying on a medical perspective.^30^ Psychosocial factors, encompassing individual and job-related aspects, influence the successful return to work.^31^

The study findings not only provide valuable insights into the relatively unexplored field of long COVID, but also hold practical implications for the development of national guidelines aimed at facilitating the treatment and management of patients with long COVID. It is crucial for future research to prioritise the role of special centres with a multidisciplinary approach in effectively managing long COVID.^32^ By building on the findings of this study and previous research, it becomes evident that various social factors extend beyond the purview of healthcare providers alone. Conducting systematic research on these factors and devising strategies to mitigate their impact on individuals could significantly contribute to the improved rehabilitation of patients with long COVID.

## Data

The dataset relied on in this article is available from the corresponding author on reasonable request.
